# Recurrent Secondary Spontaneous Pneumothorax Due to Rheumatoid Arthritis: A Report of a Rare Case

**DOI:** 10.7759/cureus.81628

**Published:** 2025-04-02

**Authors:** Vinod Kumar

**Affiliations:** 1 Pulmonary Medicine, Institute of Thoracic Medicine, Madras Medical College, Chennai, IND

**Keywords:** drugs, leflunomide, pneumothorax, recurrent, rheumatoid arthritis, spontaneous

## Abstract

Secondary spontaneous pneumothorax is typically caused by tuberculosis or emphysema. Other causes include interstitial lung disease, pneumoconiosis, organizing pneumonias, and rare conditions like Langerhans cell histiocytosis and lymphangiomyomatosis. Rheumatoid arthritis is an uncommon cause of recurrent secondary spontaneous pneumothorax due to the rupture of subpleural necrobiotic nodules. Drugs like methotrexate and leflunomide are known to accelerate the development and progression of these necrobiotic nodules. Although the exact mechanism is unknown, it is believed to result from decreased monocyte activity and increased rheumatoid factor, with macrophages acting as a nidus that interacts with rheumatoid factor to form a nodule, ultimately leading to pneumothorax when it communicates with the pleural space. This report presents a rare case of recurrent spontaneous pneumothorax resulting from the rupture of subpleural necrobiotic nodules in a patient with rheumatoid arthritis-associated lung disease.

## Introduction

Secondary spontaneous pneumothorax is typically caused by tuberculosis or chronic obstructive pulmonary disease (COPD) [1-3]. Other causes include interstitial lung disease, pneumoconiosis, malignancies, and both primary lung cancer and secondary cancers like sarcoma and sarcoidosis [1-3]. Rare causes of pneumothorax include lymphangioleiomatosis, pulmonary Langerhans cell histiocytosis, and Birt-Hogg-Dube syndrome [4,5].

Rheumatoid arthritis is a prevalent connective tissue disorder that often presents with a plethora of pleuroparenchymal complications, including pleural complications like pleural effusion, pneumothorax, empyema, and bronchopleural fistula [4,5]. Anecdotal case reports of secondary spontaneous pneumothorax due to rheumatoid arthritis [6-8] and also due to drugs used in the treatment of rheumatoid arthritis have been reported [9-11]. This case highlights the importance of considering rheumatoid arthritis-related lung involvement as a differential diagnosis in patients presenting with recurrent pneumothorax.

## Case presentation

A 46-year-old male patient, a known case of diabetes mellitus and rheumatoid arthritis for two decades, presented with a history of nausea and vomiting alongside anicteric hepatitis (alanine transaminase (ALT) and aspartate transaminase (AST) elevation to >700 units/L) one week after initiation of anti-tuberculous therapy for a clinically diagnosed pulmonary tuberculosis. Markers of viral hepatitis were negative. He was a non-smoker and non-alcoholic.

Additionally, he had a history of breathlessness and cough for six months and a history of intercostal drain insertions - once on the left hemithorax six months prior and once on the right side for pneumothorax.

General examination findings revealed telltale evidence of rheumatoid arthritis, including Jaccoud's arthropathy of the hands, fixed flexion deformity of the left elbow, and instability of the right knee due to secondary osteoarthritis, stemming from a past history of septic arthritis. Auscultation revealed bilateral wheeze.

**Figure 1:**
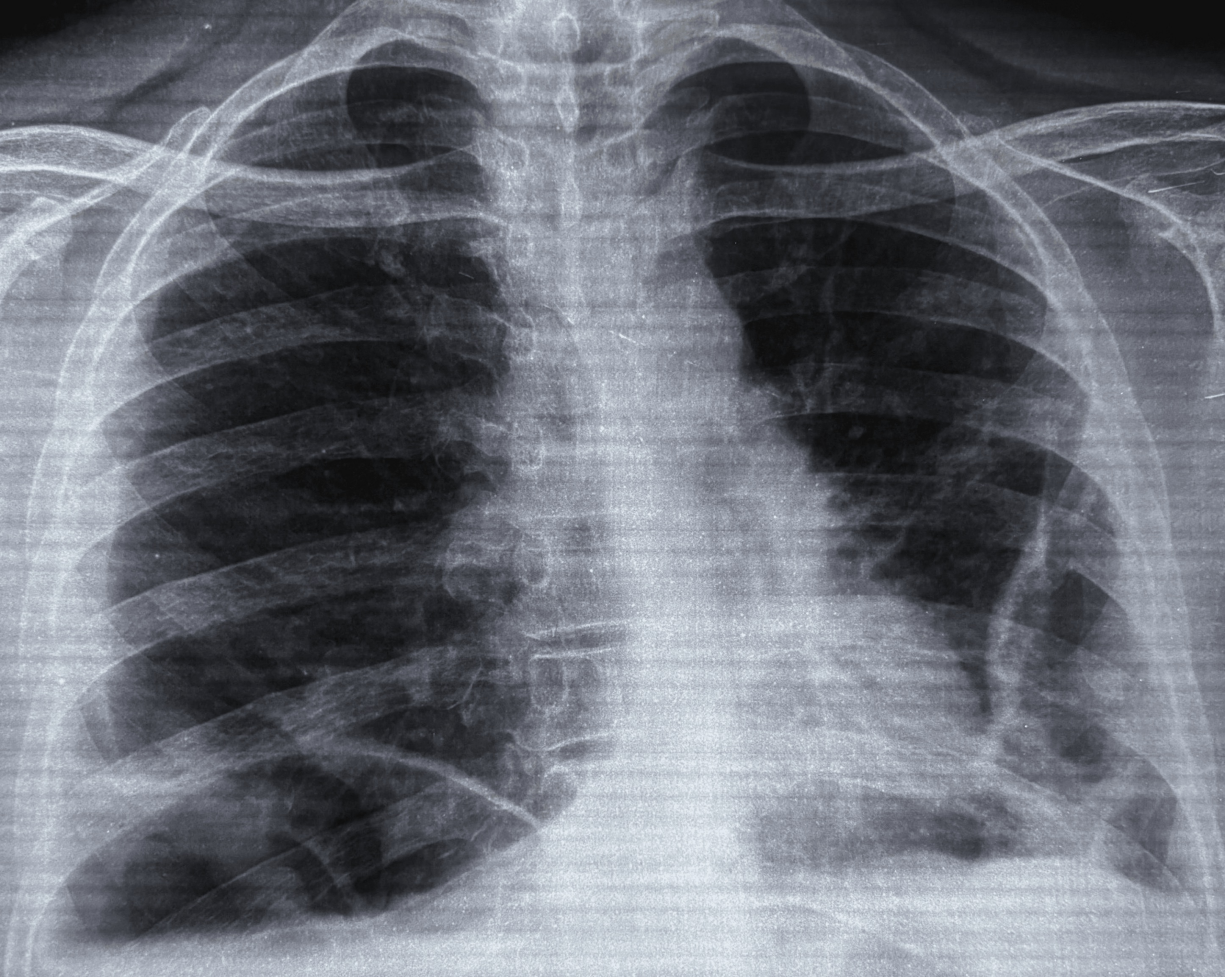
Chest X-ray of the patient showing obliteration of bilateral costophrenic angles with an air fluid level in the right lower zone

Chest X-ray (Figure 1) displayed bilateral costophrenic obliteration with an air fluid level in the right lower zone.

**Figure 2:**
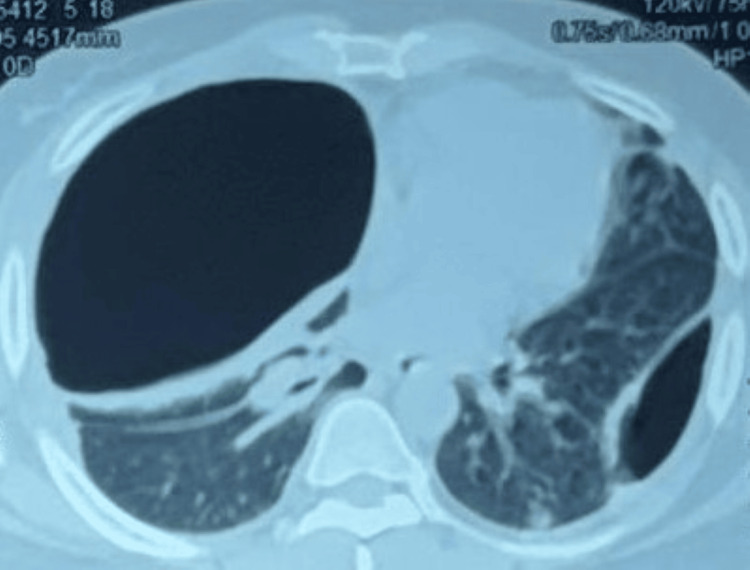
CT chest showing bilateral pneumothorax with subpleural nodules

**Figure 3:**
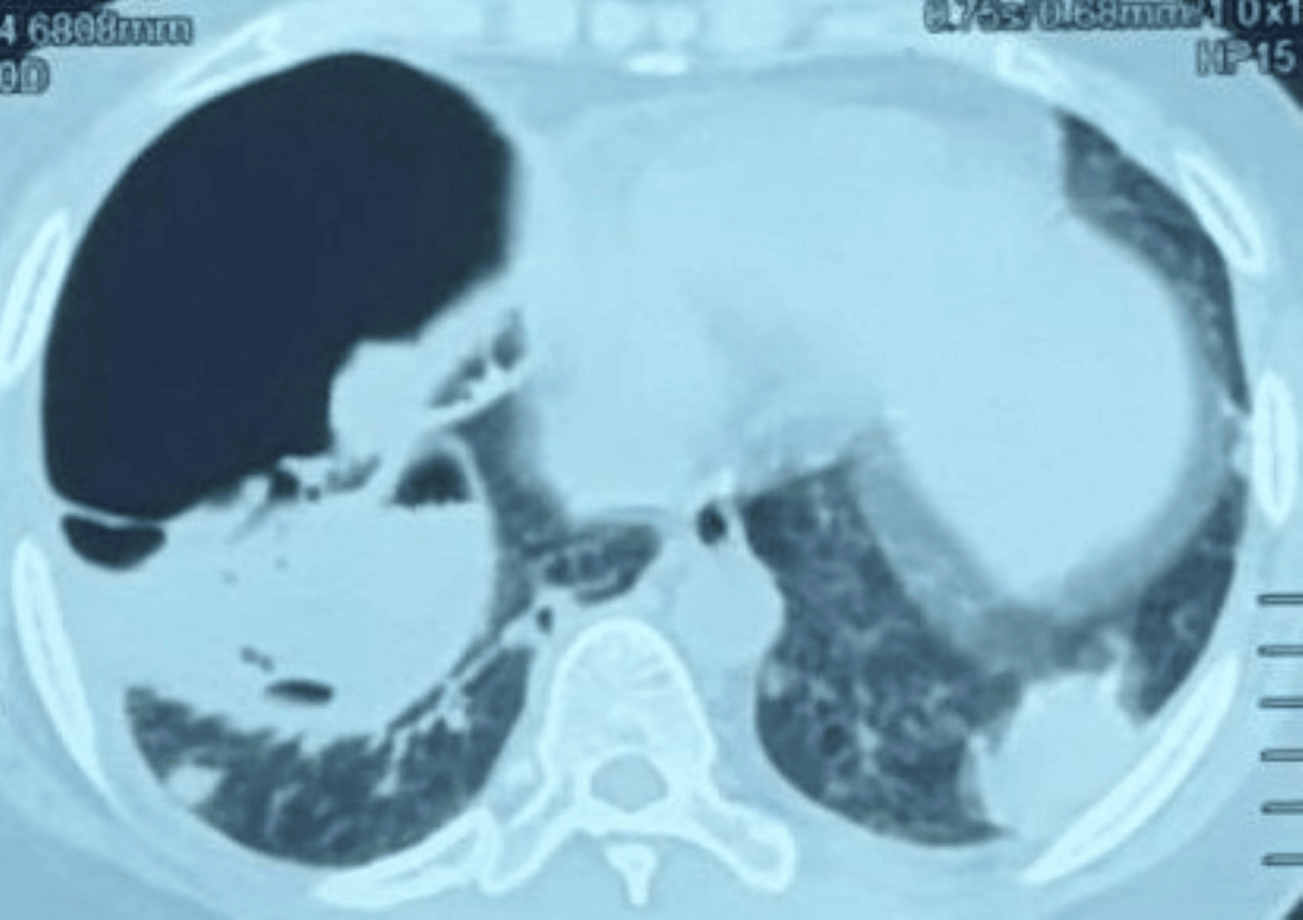
CT chest showing subpleural necrobiotic nodules

CT chest images (Figures 2-3) depicted bilateral loculated pneumothorax with multiple cavitating subpleural nodules.

Retrospectively, it was found that the patient had independently continued methotrexate 15 mg once weekly and leflunomide 10 mg daily for over four years without follow-up with his rheumatologist. A periodic review is typically advisable for patients on long-term medications to monitor drug efficacy and to identify potential complications like nephrotoxicity, hepatotoxicity, and bone marrow suppression; however, this patient had not attended any follow-up for several years. Literature indicates several reports of secondary spontaneous pneumothorax linked to rheumatoid nodules, as well as cases associated with medications used to treat rheumatoid arthritis such as methotrexate, leflunomide, etanercept, and infliximab [6-10].

The suspected offending drugs - methotrexate, leflunomide, and anti-tuberculous treatment - were stopped since his anti-tuberculous drugs were causing hepatitis, while methotrexate and leflunomide were suspected of being the causes of recurrent pneumothorax. The patient was transitioned to hydroxychloroquine in consultation with a rheumatologist. His breathlessness improved with bronchodilators, and the loculated pneumothorax was managed with oxygen therapy; he is currently on regular follow-up.

## Discussion

Secondary spontaneous pneumothorax is commonly caused by underlying lung diseases such as COPD with emphysema, tuberculosis, or interstitial lung disease, especially when associated with connective tissue disorders. In HIV patients, pneumocystis pneumonia frequently leads to secondary spontaneous pneumothorax. Rare causes include Langerhans cell histiocytosis and lymphangiomyomatosis [1-5]. Among interstitial lung diseases, pneumothorax is more commonly observed in idiopathic pulmonary fibrosis, combined pulmonary fibrosis and emphysema, and autoimmune interstitial lung disease, and is less common in non-specific interstitial pneumonia, organising pneumonia, and drug-induced interstitial lung disease [4,5].

Rheumatoid arthritis is a prevalent connective tissue disorder, and pulmonary involvement in rheumatoid arthritis is often encountered in clinical practice. Pleuro-parenchymal involvement in rheumatoid arthritis can involve the lung parenchyma, pleura, airway, and pulmonary vascular system. Parenchymal involvement encompasses interstitial lung disease, fibrobullous disease, and rheumatoid nodules. Pleural involvement can include pleural effusion, pneumothorax, empyema, and bronchopleural fistula. Airway involvement can include bronchiolitis, bronchiectasis, and cricoarytenoid arthritis. Vascular involvement can be due to pulmonary hypertension and alveolar hemorrhage. Pleuro-parenchymal involvement tends to manifest more in male patients, among those of advanced age, and in severe cases of the disease [4,5]. Our patient was a middle-aged male with long-standing rheumatoid arthritis, exhibiting deformities caused by the disease, as is typical in patients with pleuropulmonary manifestations.

Pleural involvement, with or without effusion, is the most common manifestation of rheumatoid lung disease [4,5]. Pleuritic chest pain can occur in 25% of patients with rheumatoid arthritis, and pleural effusions can be seen in 5% of such patients [5]. Spontaneous pneumothorax is uncommon in rheumatoid arthritis and is typically associated with rupture of pulmonary nodules [6-8]. Anti-rheumatic drugs like methotrexate, leflunomide, infliximab, and etanercept are known risk factors for the development of rheumatoid nodules [9,10].

Pulmonary rheumatoid nodules are well recognized but rare, occurring in less than 1% of patients [6]. Generally, pulmonary rheumatoid nodules are encountered in the presence of subcutaneous rheumatoid nodules. The histology of both subcutaneous and pulmonary nodules is the same: central fibrinoid necrosis surrounded by a palisade of radially arranged connective tissue cells, enclosed by granulation tissue (primarily lymphocytes and plasma cells) [6-8].

Reports have indicated progression and acceleration of rheumatoid necrobiotic nodules following treatment with methotrexate, leflunomide, infliximab, and etanercept [9]; rupture of these nodules into the pleural space can lead to recurrent pneumothorax, as happened in this patient. Though the exact mechanism for the development of rheumatoid nodules is unclear, the hypothesis is that there is a decrease in monocyte activity and an increase in rheumatoid factor, with macrophages acting as a nidus for rheumatoid factor to form nodules. When these nodules communicate with the pleura, they may lead to effusions, bronchopleural fistulas, or pneumothorax [10].

Our patient was started on anti-tuberculous therapy due to the presence of cavitating nodules in the lung with pneumothorax and developed hepatotoxicity, highlighting the need to raise awareness among treating physicians about the possibility of secondary spontaneous pneumothorax arising from rheumatoid nodules rather than solely from tuberculosis or emphysema. Establishing the exact cause of nodules in the lungs through bronchoalveolar lavage or image-guided biopsy may be necessary in patients lest they land with drug-induced complications due to empirical treatment of tuberculosis, as occurred in this patient. 

## Conclusions

The treating physician should be aware of the pleuroparenchymal complications associated with rheumatoid arthritis and the rupture of rheumatoid necrobiotic pulmonary nodules that can lead to pneumothorax, as well as the need to screen for these pleuroparenchymal complications. It is advisable to periodically screen patients with rheumatoid arthritis, including those undergoing treatment, for the development of lung nodules to ensure early diagnosis and management of pleuropulmonary complications. An annual low-dose CT chest, combined with lung function measurements through spirometry and a six-minute walk test, may assist in managing the pleuropulmonary complications of rheumatoid arthritis.
